# Metamaterial-enhanced infrared attenuated total reflection spectroscopy†

**DOI:** 10.1039/c8na00279g

**Published:** 2018-10-12

**Authors:** Cheng Shi, Callum Penrose, Jaqueline E. Pitts, Prarthana Gowda, Isaac J. Luxmoore, Geoffrey R. Nash

**Affiliations:** College of Engineering, Mathematics and Physical Sciences, University of Exeter Exeter EX4 4QF UK g.r.nash@exeter.ac.uk

## Abstract

The use of Fourier transform infrared spectroscopy with attenuated total reflection (FTIR-ATR) allows solid or liquid samples to be characterised directly without specific sample preparation. In such a system, the evanescent waves generated through total internal reflection within a crystal interact with the sample under test. In this work we explore the use of a mid-infrared metasurface to enhance the interaction between molecular vibrations and the evanescent waves. A complementary ring-resonator structure was patterned onto both silicon and SiO_2_/Si substrates, and the spectral properties of both devices were characterised using a FTIR-ATR system. Minima in reflectance were observed corresponding to the resonance of the metasurface on the silicon substrate, and to the hybrid resonance of phonon modes and metasurface resonances on the SiO_2_/Si substrate, in good agreement with simulations. Preliminary experiments were undertaken using mixtures containing trace amounts of butyl acetate diluted with oleic acid. Without the use of a metasurface, the minimum concentration of butyl acetate that could be clearly detected was 10%, whereas the use of the metasurface on the SiO_2_/Si substrate allowed the detection of 1% butyl acetate. This demonstrates the potential of using metasurfaces to enhance trace chemical detection in FTIR-ATR systems.

## Abstract 2

We show that a metasurface can be used in a standard FTIR-ATR instrument to enhance trace chemical detection.

## Graphical abstract

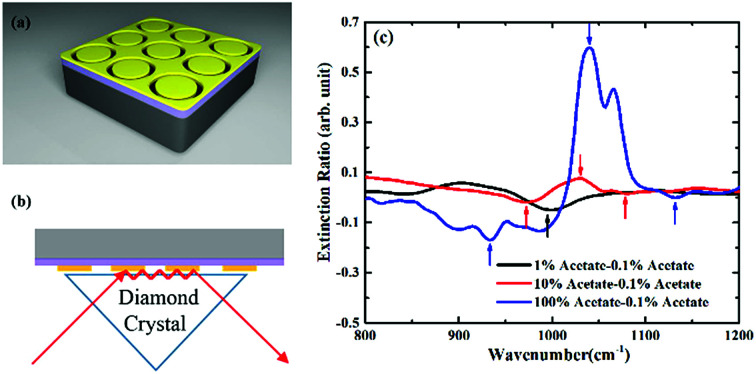

## Introduction

Infrared spectroscopy is a powerful analytical technique, providing a label-free, non-destructive method to identify chemicals.^1–3^ As the mid-infrared photon, whose energy ranges from 0.06 to 0.4 eV, can be absorbed by the unique vibrational- and rotational-modes of specific chemical bonds, infrared absorption spectra allow the identification of the chemical components of a sample by measuring the amount of IR light absorbed at specific frequencies. However, the mismatch in size between molecules and the wavelength leads to a small cross-section for light-matter interactions, and spectroscopic measurement on trace amounts of analytes remains challenging.^4^ Recent research has shown that resonant nanoparticles such as island films,^5^ arrays,^6,7^ and nano-antennas^8,9^ can be used to increase the absorption caused by molecular vibrations. The localization of the collective oscillations of electrons (plasmons) at the surface enables those designs to confine the light in a subwavelength volume with minimal energy loss,^10,11^ resulting in surface enhanced infrared absorption (SEIRA), in analogy with analogy surface-enhanced Raman scattering (SERS).

In contrast to conventional transmission or absorption spectroscopy, Fourier transform infrared spectroscopy with attenuated total reflection (FTIR-ATR) allows both liquid and solid samples to be characterised directly without specific sample preparation.^12,13^ In such a system, the evanescent waves generated through total internal reflection within a crystal interact with the sample. Although FTIR-ATR spectroscopy can achieve qualitative measurement *in situ*, its relatively poor sensitivity limits its use for quantitative analysis. One approach to improving the sensitivity of ATR is to directly attach resonant nanoparticles to ATR crystals,^14,15^ whereas Adato and Altug^16^ developed the concept of plasmonic internal reflection for biological sensing. In this manuscript we demonstrate how metasurfaces^17–21^ can be used in a standard FTIR-ATR instrument, with no modifications required, to improve the sensitivity of the measurements. As the metasurface has not been functionalized, or attached to the ATR crystal, it can also be easily re-used for many different measurements, in contrast to earlier work. Using mixtures of butyl acetate diluted with oleic acid, we show that a metasurface can be used in a standard FTIR-ATR instrument to enhance trace chemical detection, an area which has not been explored previously.

## Design and fabrication

**Fig. 1:**
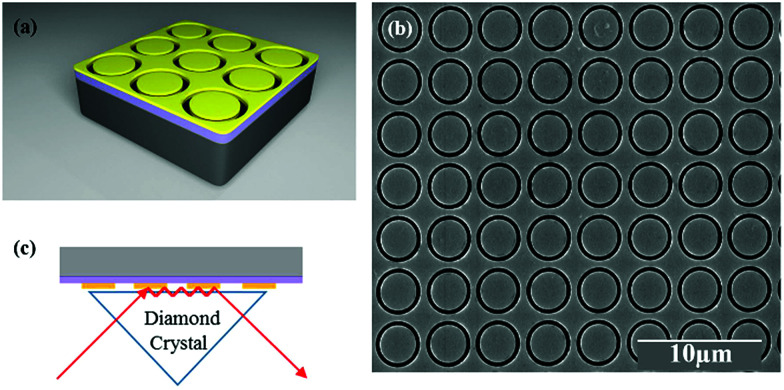
(a) Schematic diagram of the metasurface design on the SiO_2_/Si substrate; (b) top-view SEM image of a 50 nm-thick metasurface deposited on the substrate; (c) a schematic illustration of the ATR sampling technique in the measurement.

**Fig. 2:**
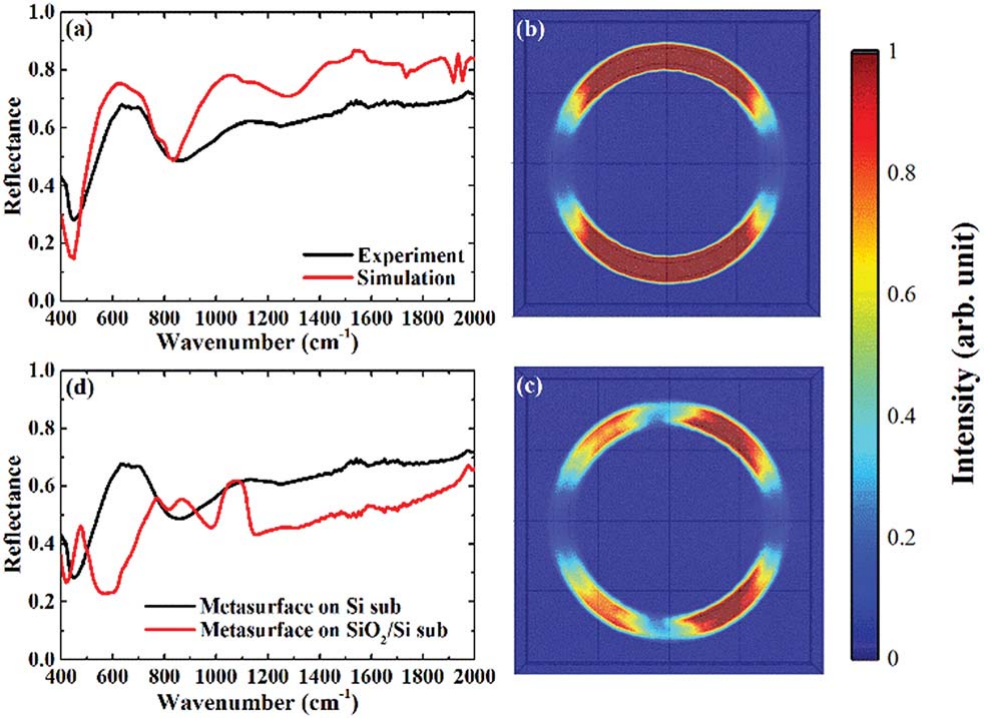
(a) Measured and simulated spectra of metasurface on Si substrate; (b) electric field distribution for *x*–*y* plane at 450 cm^−1^ (dipole mode); (c) electric field distribution for *x*–*y* plane at 860 cm^−1^ (quadrupole mode); (d) comparison between metasurface on Si substrate (black line) and on SiO_2_/Si substrate (red line).

**Fig. 3:**
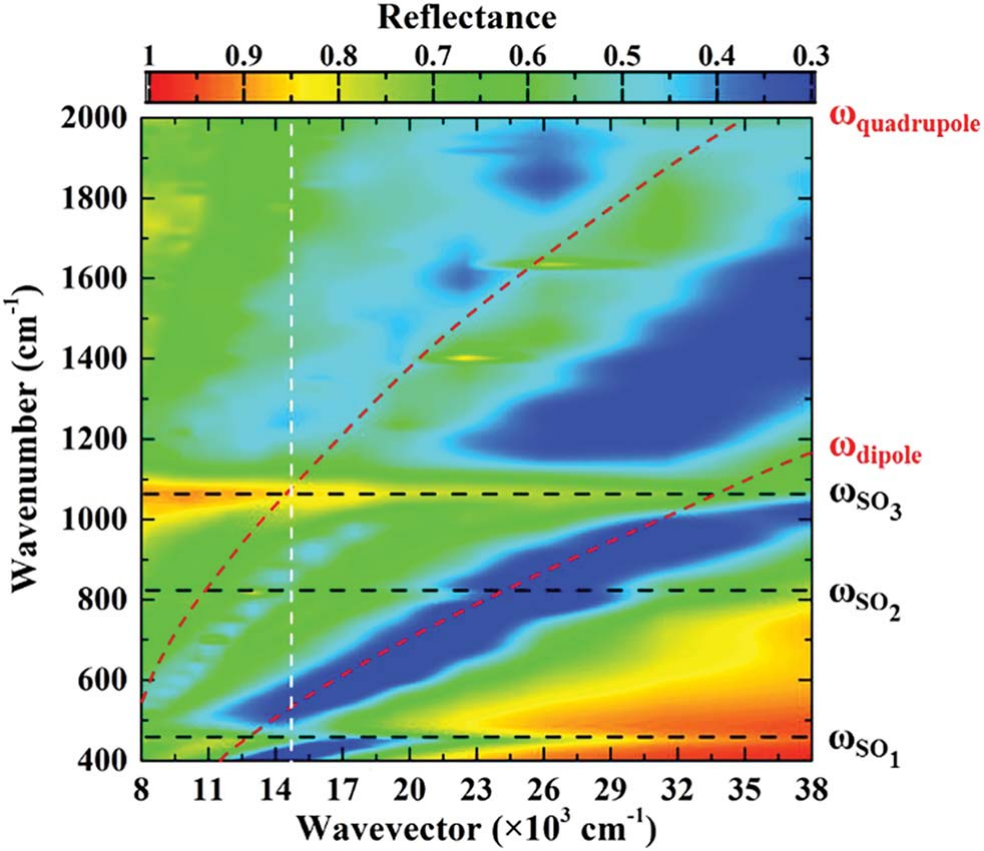
Simulated dispersion diagram of metasurface on SiO_2_/Si substrate. The two red dashed lines show the calculated resonance frequencies (dipole mode and quadrupole mode) of the uncoupled metasurface. The three black dash lines represent the frequencies of three surface optical phonon modes in SiO_2_. The vertical white line represents the spectral response of the metasurface on SiO_2_/Si substrate presented in this paper.

The metasurface consisted of an array of periodic subwavelength metallic complementary ring resonators, with a schematic of the design, and Scanning Electron Microscope (SEM) image, shown in Fig. 1(a) and (b). The inner circle of the metasurface had a radius of 1.6 μm, with each circle being isolated from the rest of the metasurface by a 300 nm wide annular gap in the gold. The periodicity in both vertical and horizontal directions was 4 μm. The coverage area of the metasurface was 2.8 mm × 2.8 mm in total, slightly larger than the ATR crystal which was used in the experiment. The metasurface is placed facing down on the ATR crystal, as shown in Fig. 1(c) (see ESI†). The measured FTIR-ATR spectrum of such a metasurface, patterned into a silicon substrate, is plotted in Fig. 2(a). There are clear minima at frequencies of 450 cm^−1^ and 860 cm^−1^ and good agreement between the measured spectrum (black line) and that calculated from using the commercial finite-element method (FEM) simulation software COMSOL (see ESI†), which is also shown in Fig. 2(a) (red line). These results confirm that the evanescent fields within the ATR crystal couple to the metasurface. The calculated electric field distributions in the plane of the surface (*x*–*y* plane) are plotted in Fig. 2(b) and show that the minima in the spectra result from different order resonances of the complementary ring resonator. The resonance at 450 cm^−1^ corresponds to the electric field being mainly confined at top and bottom of the annular gaps and forming a dipole mode. As the resonance frequency increases, the dipoles split into quadrupoles (Fig. 2(c)) and then hexapoles. Due to the oblique incident of the electromagnetic wave in the ATR system, the resonant modes also become less symmetric, and weaker in electric field confinement for higher order resonances. The same design of metasurface was also investigated when patterned into a SiO_2_/Si substrate instead of a Si only substrate, with the FTIR-ATR measured reflectance spectrum shown in Fig. 2(d). As well as slight shift of the resonances to higher frequencies, caused by the lower refractive index of the SiO_2_ layer, there are now two maxima at the resonant frequencies of dipole mode and the quadrupole mode. To identify the origin of these maxima, the dispersion of the device was calculated and is shown in Fig. 3. The dispersion clearly shows the coupling between the dipole and quadrupole resonance modes of the metasurface (red dash lines) and the surface optical (SO) phonon modes of the SiO_2_ layer^22^ at 460 cm^−1^, 805 cm^−1^ and 1084 cm^−1^ (black dash lines). It should be noted that although the Si substrate also have phonon modes at 613 cm^−1^ and 727 cm^−1^, the coupling between the metasurface and Si substrate is weaker because of the resonant frequencies of phonon modes are not close to those of the metasurface.

Compared to the metasurface resonance in the silicon based device, the hybridization of metasurface resonance and SiO_2_ phonon mode results in a much higher Q-factor, so that more energy is confined to the surface, and also higher reflectance at the resonant frequency (∼60%).

## Results

**Fig. 4:**
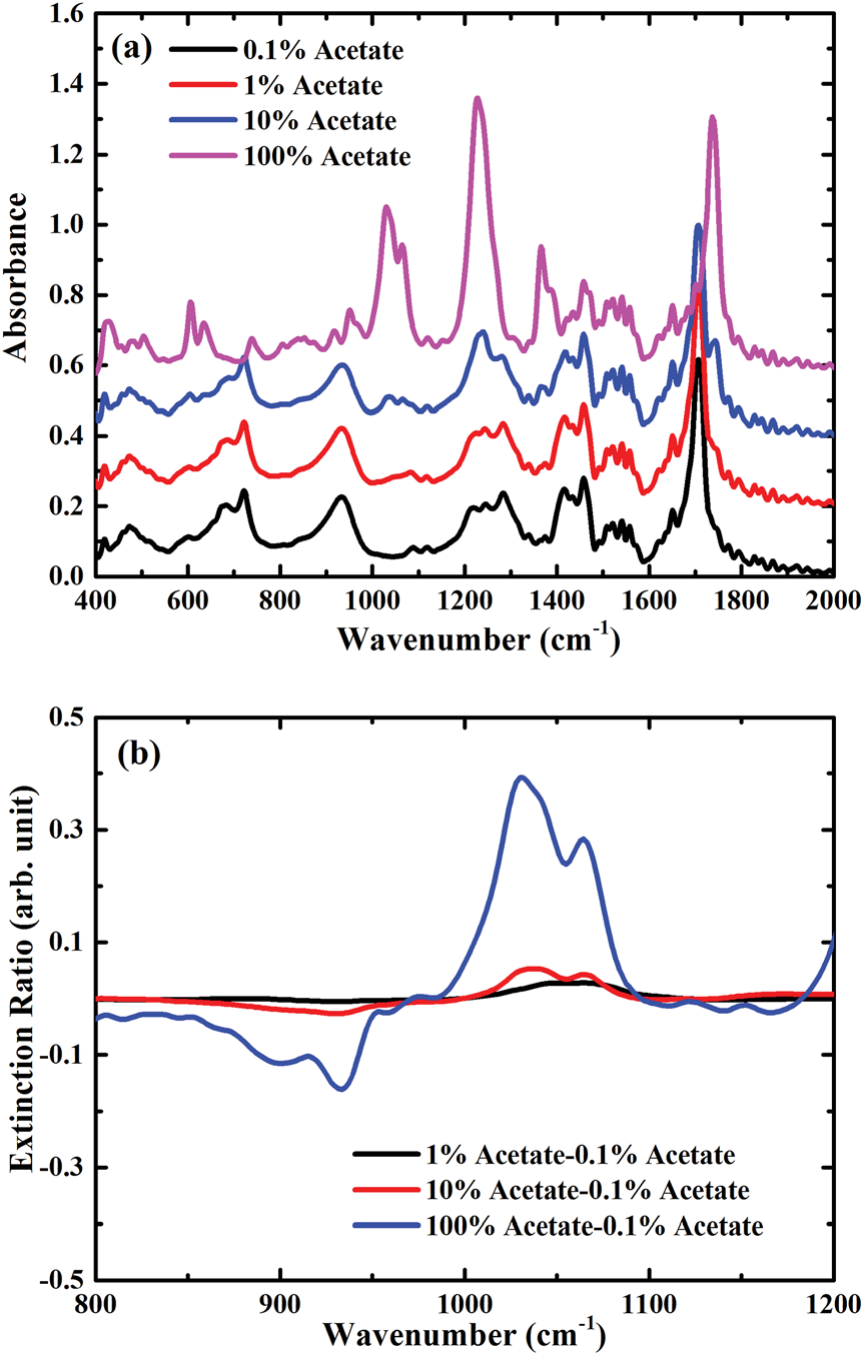
(a) Measured absorption spectra of mixtures only in the ambient atmosphere, spectra are consecutively shifted by 0.2 vertically for clarity; (b) zoomed extinction spectra of mixtures only for the carbon–oxygen stretching band region between 800 cm^−1^ and 1200 cm^−1^.

To demonstrate the feasibility of using such a metasurface to enhance the sensitivity of FTIR-ATR spectroscopy, preliminary measurements were undertaken using mixtures of butyl acetate and oleic acid (see ESI†), which are two important constituents of many foods, for example olive oil.^23,24^ In Fig. 4(a) FTIR-ATR spectra are shown for different mixtures of butyl acetate and oleic acid. All the spectra contain a large number of maxima in the absorption corresponding to the excitation of vibrational resonances.^3^ The measurements taken with FTIR-ATR solely demonstrates the current limitation of infrared spectroscopy in detecting trace chemicals. For example, the infrared spectrum of pure butyl acetate contains a strong maxima in absorption at a frequency of approximately 1050 cm^−1^, corresponding to one of the C–O stretching bands associated with an ester,^3^ which is not present in the spectrum of oleic acid. The size of this absorption feature can therefore be used as a way of determining the amount of butyl acetate present in a mixture of butyl acetate and oleic acid. However, this maximum in absorption is only just discernible in the mixture containing 10% butyl acetate. This is confirmed by plotting the extinction ratio, Fig. 4(b), which is defined as (*A*_mix_ − *A*_0.1%_)/*A*_0.1%_, where *A*_mix_ is the absorbance of the mixture sample, and *A*_0.1%_ the absorbance of the 0.1% dilution. Whilst strong absorption is seen in the pure butyl acetate extinction ratio at 1050 cm^−1^, the same feature is approximately 8× smaller for the mixture containing 10% butyl acetate, demonstrating the limit of sensitivity of this kind of measurement.

**Fig. 5:**
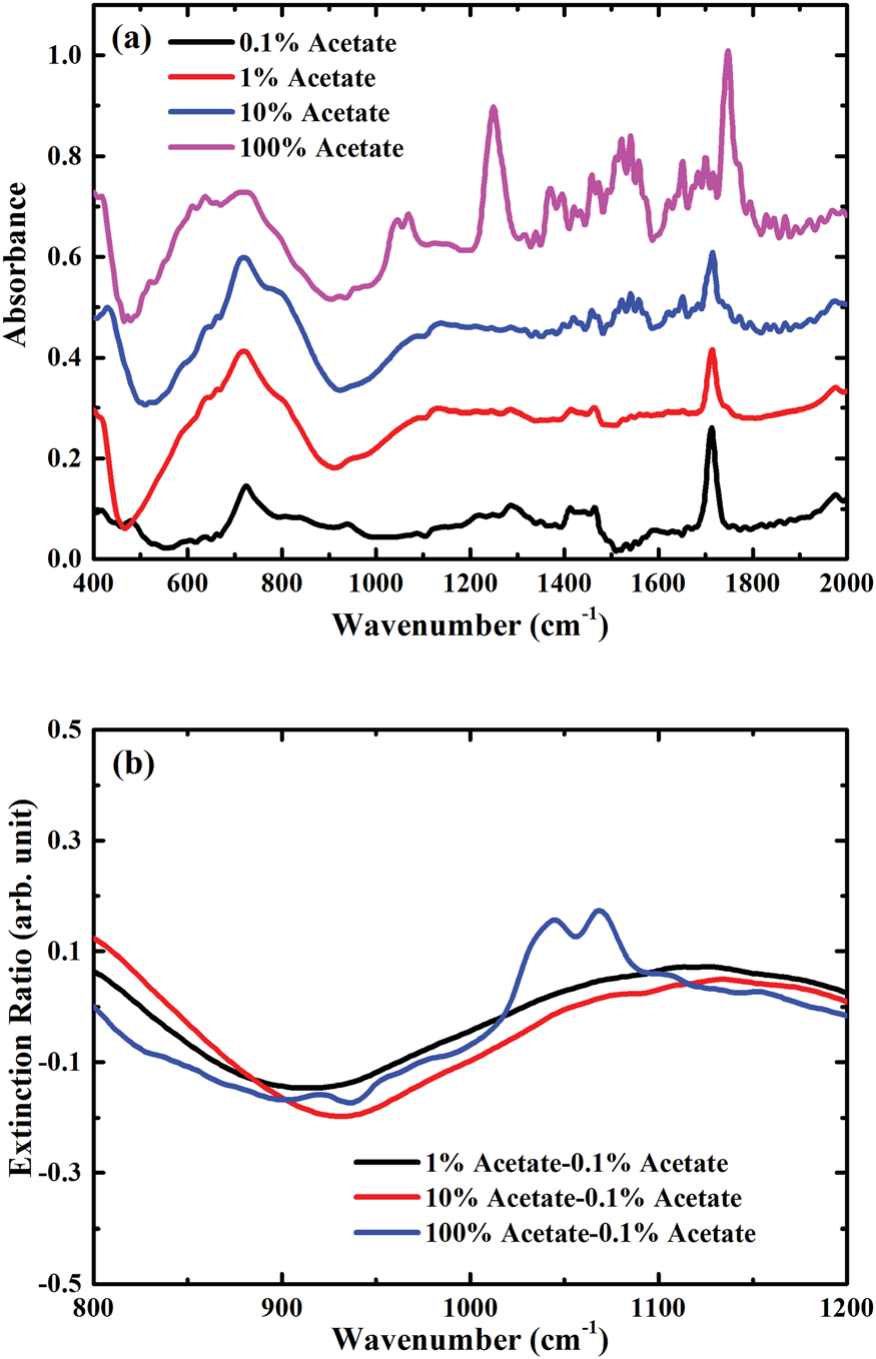
(a) Measured absorption spectra of mixtures in the presence of metasurface on Si substrate in the ambient atmosphere, spectra are consecutively shifted by 0.2 vertically for clarity; (b) zoomed extinction spectra of mixtures in the presence of metasurface on Si substrate for the carbon–oxygen stretching band region between 800 cm^−1^ and 1200 cm^−1^.

To explore whether the use of a metasurface can be used to improve the ability to detect trace chemicals, the same dilutions were measured, but with the metasurface face down on top of the analyte. In Fig. 5(a) the FTIR-ATR spectra are plotted for different chemical dilutions with the metasurface on a silicon substrate. In addition to the fingerprint absorption peaks described above, the two main minima associated with the metasurface, at frequencies of approximately 450 cm^−1^ and 870 cm^−1^, are clearly seen (note that for the metasurface results, the spectra are normalised to the measured spectra of the metasurface without dilutions). This confirms that the evanescent waves within the ATR crystal are able to excite the metasurface resonances, even in the presence of the analyte. These two resonances correspond to the dipole and quadrupole resonance modes of the metasurface, which shifts with increasing concentration of butyl acetate. In this case, as the refractive index of butyl acetate is higher than that of oleic oil, the effective refractive index of the metasurface increases with the concentration of butyl acetate, resulting in the observed shift of the metasurface resonances, as shown in Fig. 5(b). However, the relative broadness of the metasurface resonances, and the fact that there is a resonance at 950 cm^−1^ rather than at 1100 cm^−1^, means that it is still not possible to detect the presence of 1% or 10% butyl acetate in oleic acid. The extinction ratio, shown in Fig. 5(b), confirms that the shape of the absorption at ∼1050 cm^−1^ does not significantly change as the concentration of butyl acetate is decreased.

**Fig. 6:**
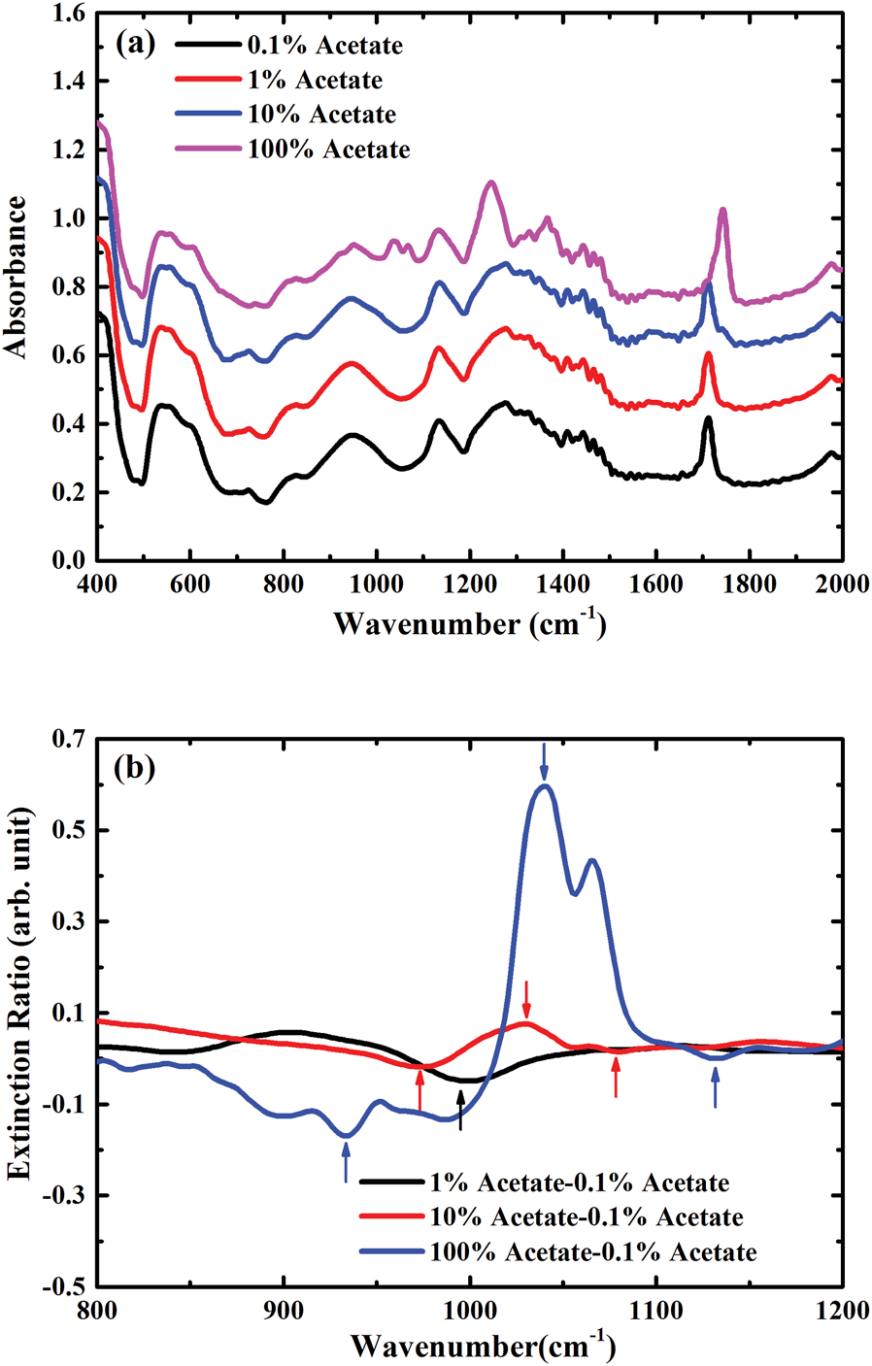
(a) Measured absorption spectra of mixtures in the presence of metasurface on SiO_2_/Si substrate in the ambient atmosphere, spectra are consecutively shifted by 0.2 vertically for clarity; (b) zoomed extinction spectra of mixtures in the presence of metasurface on SiO_2_/Si substrate for the carbon–oxygen stretching band region between 800 cm^−1^ and 1200 cm^−1^.

For the metasurface on a SiO_2_/Si substrate, there is much more overlap between one of the resonances of the metasurface, as shown in Fig. 2(d), and the vibrational modes in butyl acetate. This overlap has a strong effect on the measured FTIR-ATR spectra for the different mixtures on the metasurface, which are plotted on Fig. 6(a). In this case, even in the mixture containing only 1% butyl acetate, a clear maximum is seen in the measured spectra at around 1050 cm^−1^. The plot of the extinction ratio, Fig. 6(b), shows how the shape of the spectra now changes with the concentration of butyl acetate, indicative of coupling between the vibrational resonances (dark mode) with the hybridised metamaterial phonon mode (bright mode). As the concentration of acetate increases, the minimum seen at ∼1000 cm^−1^ (marked by a black arrow) splits into two minima (marked by up arrows in red) and form a maxima in the middle (marked by a down arrow in red). The coupling become even stronger for pure butyl acetate (see blue arrows), and it should also be noticed that the extinction ratio is also increased due to the coupling. Further work is now underway to optimise the design of the metasurface to further enhance this coupling.

## Conclusions

In this work, we explore the use of a mid-infrared metasurface to enhance the interaction between molecular vibrations and the evanescent waves within an ATR crystal. A complementary ring-resonator structure was patterned onto both silicon and SiO_2_/Si substrates, and the spectral properties of both devices were characterised using a FTIR-ATR system. Resonant features were observed corresponding to the resonance of the metasurface on the silicon substrate device, and the hybrid resonance of phonon modes and metasurface resonance on the SiO_2_/Si substrate, in good agreement with simulations. Preliminary experiments were undertaken using mixtures of butyl acetate diluted with oleic acid. Without the use of a metasurface, the minimum concentration of butyl acetate that could be detected was 10%, whereas the use of the metasurface on the SiO_2_/Si substrate allowed the detection of 1%, due to the coupling of the vibrational resonances of the butyl acetate with the hybridised metamaterial phonon mode. These results demonstrate the potential of the use of metamaterials to improve the sensitivity of FTIR-ATR measurements, and offers a new route for improved trace chemical detection.

## Conflicts of interest

There are no conflicts to declare.

## Supplementary Material

NA-001-C8NA00279G-s001
