# Supplementary material for: Metamaterial-enhanced infrared attenuated total reflection spectroscopy
Source: Nanoscale Adv. 2018 Oct 12;1(2):476–80. doi: 10.1039/c8na00279g (PMC9473245; doi:10.1039/c8na00279g)
Supplement: NA-001-C8NA00279G-s001 [file NA-001-C8NA00279G-s001.pdf]

Electronic Supplementary Material (ESI) for Nanoscale Advances.
This journal is © The Royal Society of Chemistry 2018

Supporting Information

Metamaterial-enhanced Infrared Attenuated Total Reflection Spectroscopy

Cheng Shi,[a] Callum Penrose,[a] Jaqueline E. Pitts,[a] Prarthana Gowda,[a] Isaac J. Luxmoore,[a] Geoffrey R. Nash [a]

[a.] *College of Engineering, Mathematics and Physical Sciences, University of Exeter, Exeter, EX4 4QF, United Kingdom.*

This supplementary information includes:

1. Simulation Section
2. Sample Fabrication Section
3. Experiment Setup Section
4. Analytes Preparation Section
5. Supplemental Figures
6. Supplemental Reference

Simulation

The Radio Frequency (RF) Module in Comsol Multiphysics was selected for our simulation. The unit cell of sample contains a dielectric layer with refractive index equals to 2.53 as the diamond crystal, beneath which is the unit cell of the metasurface and the substrate. For the SiO_2/Si substrate, a 300nm thick SiO_2 layer is added between the metasurface the Si substrate. Since the thickness of the diamond crystal and silicon substrate is much larger than the maximum wavelength, Perfect Matching Layer (PML) conditions are all applied at z-axis whilst the boundary conditions for x-axis and y-axis are defined as periodic. The wave source is defined as a periodic port with angle of incidence equals to 45 ˚. The permittivity of Si and SiO_2 are modelled by using Lorentz model, as used in our previous researches [1].

Sample Fabrication

The substrate we chose for the device is non-doped silicon (ϱ =3000ohm·cm) and 300nm thermal oxidized SiO_2 layer on top of the non-doped silicon , which both have relatively low absorption in the mid-infrared regime. Polymethylmethacrylate diluted in 6% anisole (PMMA 950K A6) was spun on the top of the substrate with 4000rpm as the resist for lithography and baked at 150°C for 10min to remove the solvent. The resist is then patterned by Electron Beam Lithography (NanoBeam nB4) and developed in a 3:1 ratio of isopropyl alcohol (IPA), methyl iso-butyl ketone (MIBK) solution before metallisation. 5 nm of Cr was deposited initially followed by 50 nm of Au on top of the sample. The unwanted metal and PMMA were removed by dissolving the PMMA in warm acetone at 50 °C followed by IPA. An ultrasonicator was used to aid in the lift off of the metamaterial structures.

Experiment Setup

Fourier Transform Infrared Spectroscopy (Bruker Vertex 80v) in conjunction with Attenuated Total Reflection module (Platinum ATR Accessory) was used to characterize the biological samples in the experiment. The infrared wave sourced from the internal mid-IR Globar source through the interoferometer injects on the diamond prism over the critical angle, the generated evanescent wave interacts with the meta-device and chemical dilution and then the final signal is collected by the liquid nitrogen cooling Cadmium Mercury Telluride (CMT) detector. Spectra were recorded with 8cm^{-1} wavenumber resolution in the wavenumber range from 2000 to 400 cm^{-1} that are averaged over 256 scans per measurement. All samples were placed on top of the ATR spectrometer with the metasurface side of the device always facing the diamond crystal.

Analytes Preparation

Both butyl acetate (purity ≥ 99%) and oleic oil (purity ≥ 99%) used in the paper were purchased from Sigma Aldrich. The mixture analytes used in the characterization are butyl acetate diluted with oleic acid, the descriptions state the butyl acetate percentage in the liquid (e.g. 1% Acetate means 1% butyl acetate and the other 99% is oleic acid, by volume). The thickness of liquid was controlled by using the same volume (0.5μL) of liquid and applying an equal contact pressure on each device or substrate used. Using the measured absorption spectra, the imaginary part of the refractive index, κ, of the mixtures was calculated from the Lambert-Beer Law, using the method introduced by Rowe et al [2]. The real part of the refractive index, n, can also be deduced from the imaginary part by using Kramers-Kronig (KK) transform [3] (Figure S1). In order to ensure the thicknesses of analytes on the metasurface are approximately the same in every measurement, thickness calibrations were undertaken for all the results based on retrieved refractive indices.

Supplemental Figures

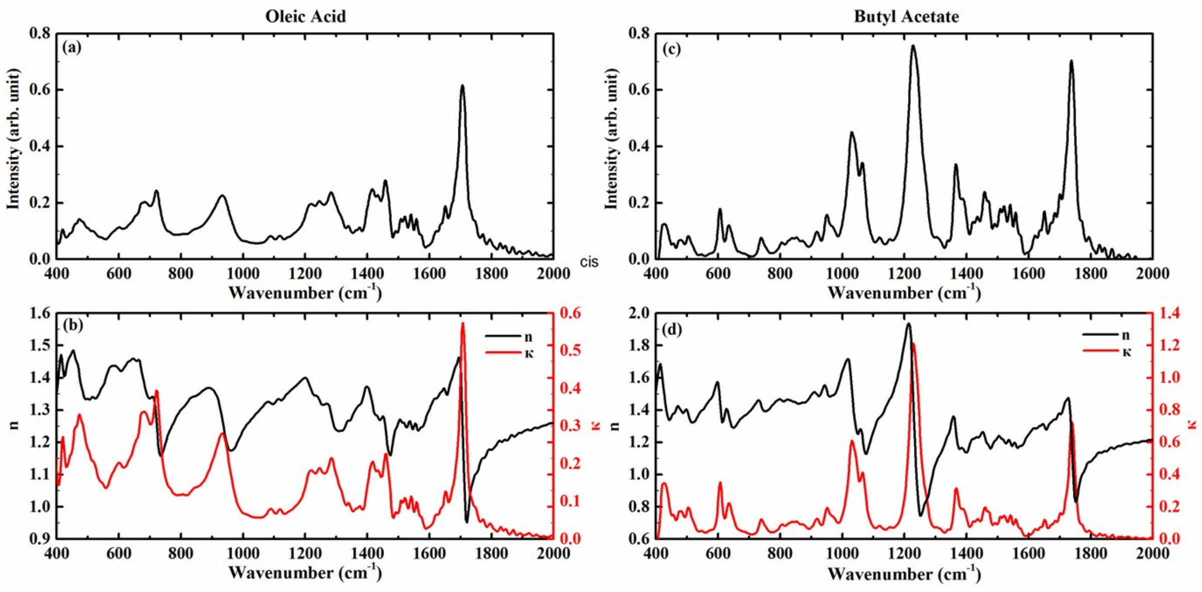

Figure S1 (a) Measured Absorption spectrum and (b) calculated refractive index of Oleic Acid; (c) Measured Absorption spectrum and (d) calculated refractive index of Butyl Acetate

Supplemental Reference

1 P. Q. Liu, I. J. Luxmoore, S. A. Mikhailov, N. A. Savostianova, F. Valmorra, J. Faist, G. R. Nash, *Nat. Comm.*, 2015, **6**, 8969.

2 D. J. Rowe, D. Smith, J. S. Wilkinson, *Sci. Rep.*, 2017, **7**(1), 7356.

3 V. Lucarini, J. J. Saarinen, K. E. Peiponen, E. M. Vartiainen, *Kramers-Kronig Relations in Optical Materials Research*, Springer, 2005.
